# Electrostatic Origins of CO_2_-Increased Hydrophilicity in Carbonate Reservoirs

**DOI:** 10.1038/s41598-018-35878-3

**Published:** 2018-12-06

**Authors:** Yongqiang Chen, Ahmad Sari, Quan Xie, Patrick V. Brady, Md Mofazzal Hossain, Ali Saeedi

**Affiliations:** 1 Department of Petroleum Engineering, Curtin University, 26 Dick Perry Avenue, 6151 Kensington, Western Australia Australia; 2 Sandia National Laboratories, Albuquerque, New Mexico 87185-0754 United States

## Abstract

Injecting CO_2_ into oil reservoirs appears to be cost-effective and environmentally friendly due to decreasing the use of chemicals and cutting back on the greenhouse gas emission released. However, there is a pressing need for new algorithms to characterize oil/brine/rock system wettability, thus better predict and manage CO_2_ geological storage and enhanced oil recovery in oil reservoirs. We coupled surface complexation/CO_2_ and calcite dissolution model, and accurately predicted measured oil-on-calcite contact angles in NaCl and CaCl_2_ solutions with and without CO_2_. Contact angles decreased in carbonated water indicating increased hydrophilicity under carbonation. Lowered salinity increased hydrophilicity as did Ca^2+^. Hydrophilicity correlates with independently calculated oil-calcite electrostatic bridging. The link between the two may be used to better implement CO_2_ EOR in fields.

## Introduction

Oil will be an important energy source for the rest of the 21^st^ century^1^ and carbonate reservoirs host most of the world’s oil (>60%)^2^. However, the recovery factor is low (<40%)^3^, so there is enormous motivation to improve recovery cost-effectively, and with environmentally friendly manners. CO_2_ EOR is attractive because it produces more oil without the expense of chemicals although CO_2_ injection costs energy to compress before injection, and the CO_2_ source availability needs to be also considered. Moreover, CO_2_-EOR can combat global warming by injecting CO_2_ into geological formations. CO_2_ EOR techniques include miscible^4^ and immiscible continuous injection^5,6^, carbonated water flooding^7^, huff and puff injection (injecting CO_2_ in a single well and producing from the well after CO_2_ equilibration with the crude oil)^8,9^, and water-alternating-CO_2_ injection^10–12^. CO_2_ techniques work through some combination of immiscible drive, first contact miscible drive, vaporizing-gas drive, condensing-gas drive, and vaporizing-condensing gas drive, and multiple-contact miscible drive. At the microscopic level, these processes can: promote oil-swelling, reduce oil viscosity, mitigate gravity segregation by reducing the density difference between oil and water, and, lower oil interfacial tension, all of which can increase oil recovery. The net impact of CO_2_ addition can be quite large, amounting to recovery of an extra 4–15% of the original oil in place in conventional reservoirs^13^. Moreover, CO_2_ huff-n-puff can achieve 14% additional oil recovery from unconventional reservoirs^14^.

While much is known about the effect of CO_2_ on oil fluid properties, oil-CO_2_-brine-carbonate system wettability is not well understood, which triggers intrinsic uncertainties to predict and manage the CO_2_ injection and reservoir performance although CO_2_-brine-rock system wettability has been well documented^15,16^. This is largely because system wettability governs subsurface multiphase flow and residual saturations^17^. To examine the wettability, contact angle test has been perceived as an effective means together with interpretation using Derjaguin-Landau-Verwey-Overbeek (DLVO)^18–20^ and surface complexation modelling^21–23^.

Teklu *et al*.^10^ showed that dissolving CO_2_ into seawater decreases oil contact angles on calcite, thus increasing hydrophilicity. Decreased salinity also decreases contact angles. Teklu *et al*.^10^ noted several potential explanations for their contact angle trends and called for a closer examination of the surface controls over wettability alteration. Venkatraman *et al*.^24^ used Gibbs free-energy function to integrate phase-behaviour computations and geochemical reactions to find equilibrium composition, but quantitative work remains to be made to understand how dissolved CO_2_ governs oil-brine-calcite interaction, thus wettability. Here we constrain surface chemical controls over wettability in carbonate reservoirs undergoing CO_2_ EOR by interpreting new oil-on-calcite contact angles in the presence of model reservoir brines containing NaCl and CaCl_2_ using a coupled surface complexation/CO_2_ and mineral dissolution model.

## Results and Discussion

**Figure 1:**
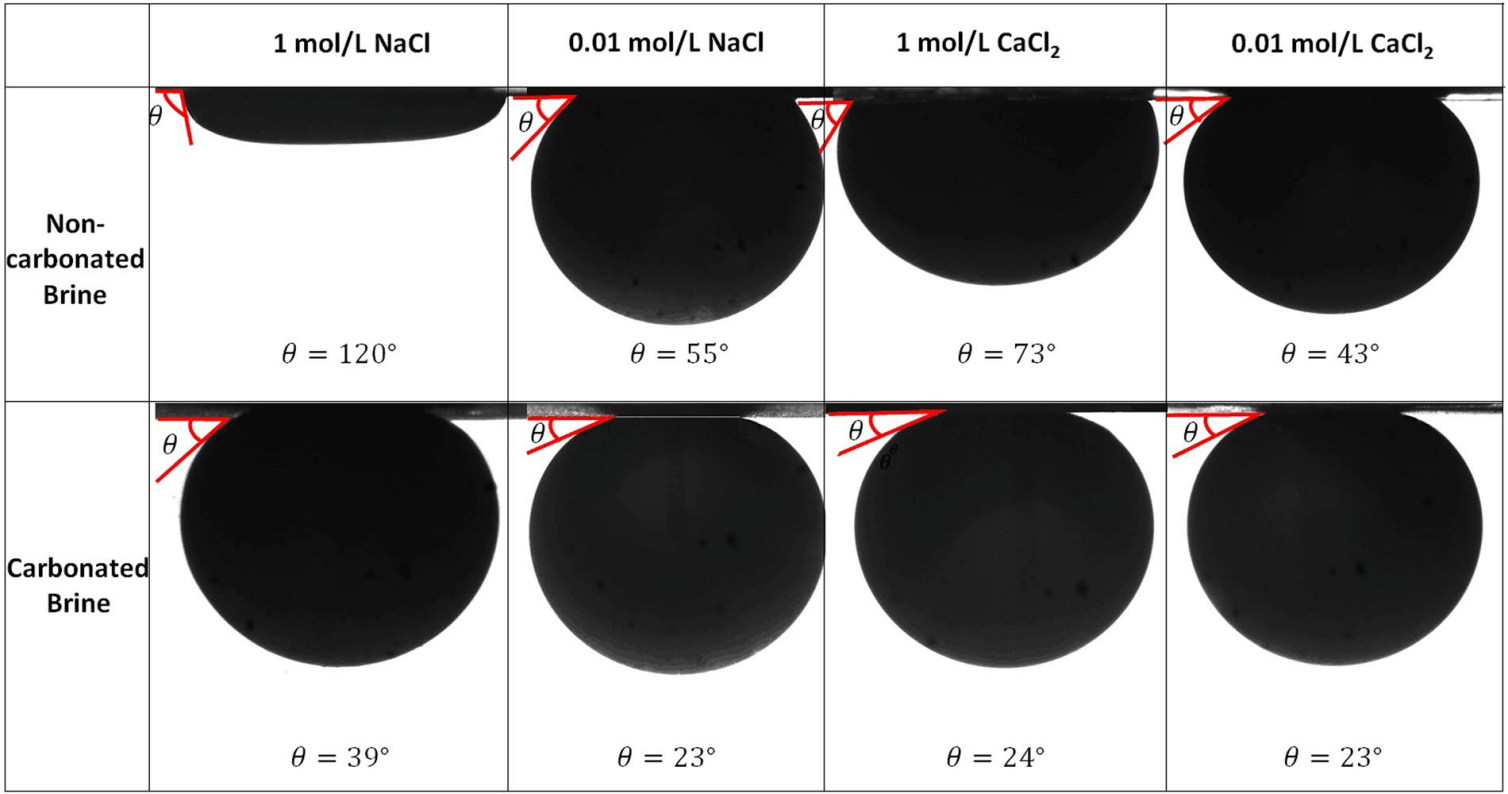
Oil-on-calcite contact angles in the presence of carbonated and non-carbonated brines. In total, we measured eight contact angles, four contact angles with carbonated brine and four contact angles with non-carbonated brine. The standard deviation of contact angle measurements was ±2°.

To examine the wettability of oil-brine (CO_2_)-carbonate system, we measured contact angle of oil on calcite substrates in the presence of carbonated brine or non-carbonated brine. Figure 1 shows oil-on-calcite contact angles measured at 25 °C and 3000 psi pressure in model brines under carbonated and non-carbonated conditions. Carbonated water lowers contact angles and produces a strongly water-wet system regardless of salinity and ion type compared to non-carbonated water. For example, non-carbonated 1 mol/L NaCl yielded a contact angle of 120°, meaning an oil-wet system. However, carbonated 1 mol/L NaCl gave a contact angle of 39°, meaning a strongly water-wet system. Similarly, Teklu *et al*.^10^ observed a contact angle shift from 116.6–133.6° (non-carbonated seawater, pH = 6.6) to 36.1–40.8° (carbonated seawater, pH = 5.5 at atmospheric condition). A secondary effect of lowered salinity decreasing contact angles and moving the system towards water wetness is also seen in Fig. 1, and was observed before by Teklu *et al*.^10^. Divalent cations (Ca^2+^) gave a lower contact angle compared to monovalent cations (Na^+^) regardless of concentration.

**Figure 2:**
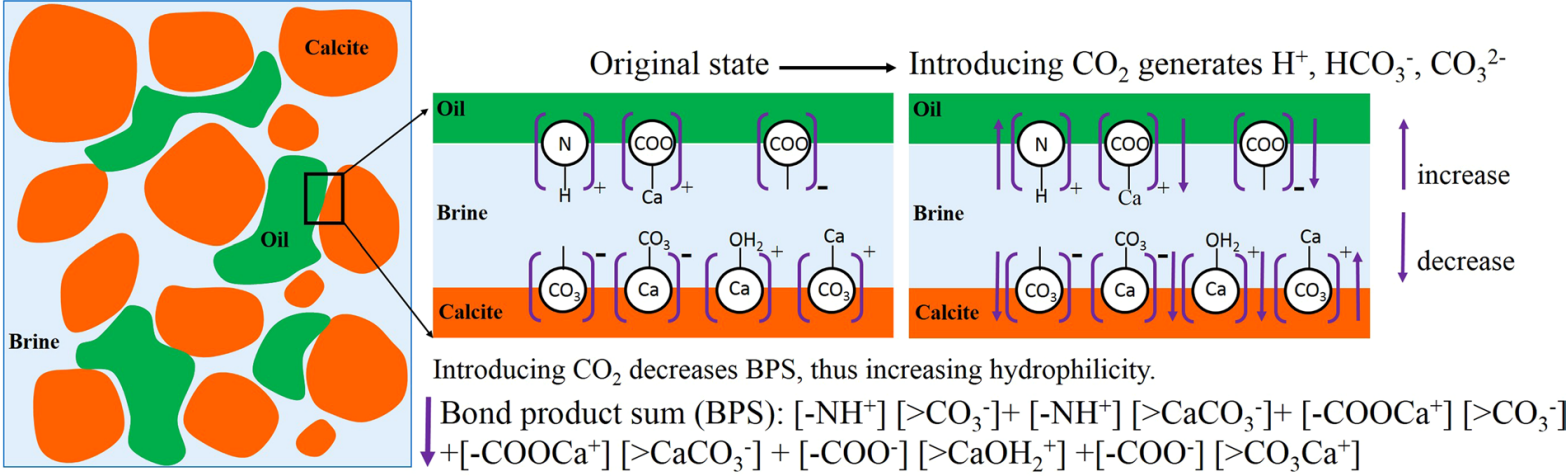
Schematic of surface chemistry alteration during CO_2_ EOR.

To understand how carbonation increases hydrophilicity, we develop a geochemical model that couples CO_2_ dissolution, mineral dissolution, and oil and calcite surface chemistry (Table 1). CO_2_ and calcite dissolution into brines is calculated by a standard equilibrium approach. Oil surface species are assumed to be –NH^+^, –COO^−^ and –COOCa^+^ ^23,25,26^, polar surface groups expressed at, and attached to, the oil-water interface. Calcite surface species are assumed to be >CO_3_^−^, >CaCO_3_^−^, >CaOH_2_^+^, and >CO_3_Ca^+^ ^21–23,27^ (Fig. 2); where “>” denotes a calcite surface species. The primary electrostatic bridges between oppositely charged oil and calcite surface species are then the pairs, -NH^+^ and >CO_3_^−^, -NH^+^ and >CaCO_3_^−^, -COOCa^+^ and >CO_3_^−^, -COOCa^+^ and >CaCO_3_^−^, -COO^−^ and >CaOH_2_^+^, and -COO^−^ and> CO_3_Ca^+^. A quantitative measure of electrostatic attraction is termed the bond product sum^21,23^, BPS, which is equal to [-NH^+^] [>CO_3_^−^] + [-NH^+^] [>CaCO_3_^−^] + [-COOCa^+^] [>CO_3_^−^] + [-COOCa^+^] [>CaCO_3_^−^] + [-COO^−^] [>CaOH_2_^+^] + [-COO^−^] [>CO_3_Ca^+^]; where bracketed terms are calculated surface concentrations (μmol/m^2^). Bond product sum (electrostatic bridges) is an explicit way to reflect the electrostatic force change between the oil/brine and rock/brine interfaces. Our previous studies^25,28^ show that DLVO and surface complexation modelling predict similar wettability trends. This is because the physics of DLVO and surface complexation is the same as a result of diffuse double layer. We therefore decided to use BPS to reveal the interaction of oil-brine-carbonate because BPS can be practically modelled using the geochemical reactions with reservoir simulators for waterflooding and EOR studies.

**Table 1:** Surface complexation model input parameters.

Interfaces	Reaction	Log K_25_ °C	Reaction
Oil/Brine Interface	-NH^+^ = -N + H^+^	−6.0	1
	-COOH = –COO^−^ + H^+^	−5.0	2
	-COOH + Ca^2+^ = -COOCa^+^ + H^+^	−3.8	3
Calcite/Brine Interface	>CaOH + H^+^ = >CaOH_2_^+^	11.85	4
	>CaOH + HCO_3_^−^ = >CaCO_3_^−^ + H_2_O	5.8	5
	>CaOH_2_^+^ + SO_4_^2−^ = >CaSO_4_^−^ + H_2_O	2.1	6
	>CO_3_H = >CO_3_^−^ + H^+^	−5.1	7
	>CO_3_H + Ca^2+^ = >CO_3_ Ca^+^ + H^+^	−2.6	8
	>CO_3_H + Mg^2+^ = >CO_3_ Mg^+^ + H^+^	−2.6	9

“>” represents the negatively charged site on the carbonate surface while the “−” represents the negatively charged site on the oil surface. Given that directly sorbed oil probably doesn’t respond to low salinity waterflooding, and that only oil-rock with an intervening water layer will respond^23^, our analysis focusses solely on the water-present situation which can be modelled using surface complexation theory^23,29^. In our geochemical modelling, we did not consider the interaction between non-polar oil and calcite surfaces, e.g., hydrogen bonding, Van der Waals interaction, and ligand bridging, etc.^45^. However, we can reasonably assume that acidic and amine functional groups governs the electrostatic surface species at oil surfaces, which dominates the adhesion force between oil and rock surfaces^46–48^. Moreover, water assisted EOR (e.g., carbonated water and low salinity water) plays a main role in the interaction of polar part and rock surfaces^46,49,50^, but the interaction between non-polar oil and calcite surfaces plays a little effect in water assisted EOR^51,52^. Our assumption therefore can be reasonably justified.

### Speciation of Oil/Brine Interfaces

**Figure 3:**
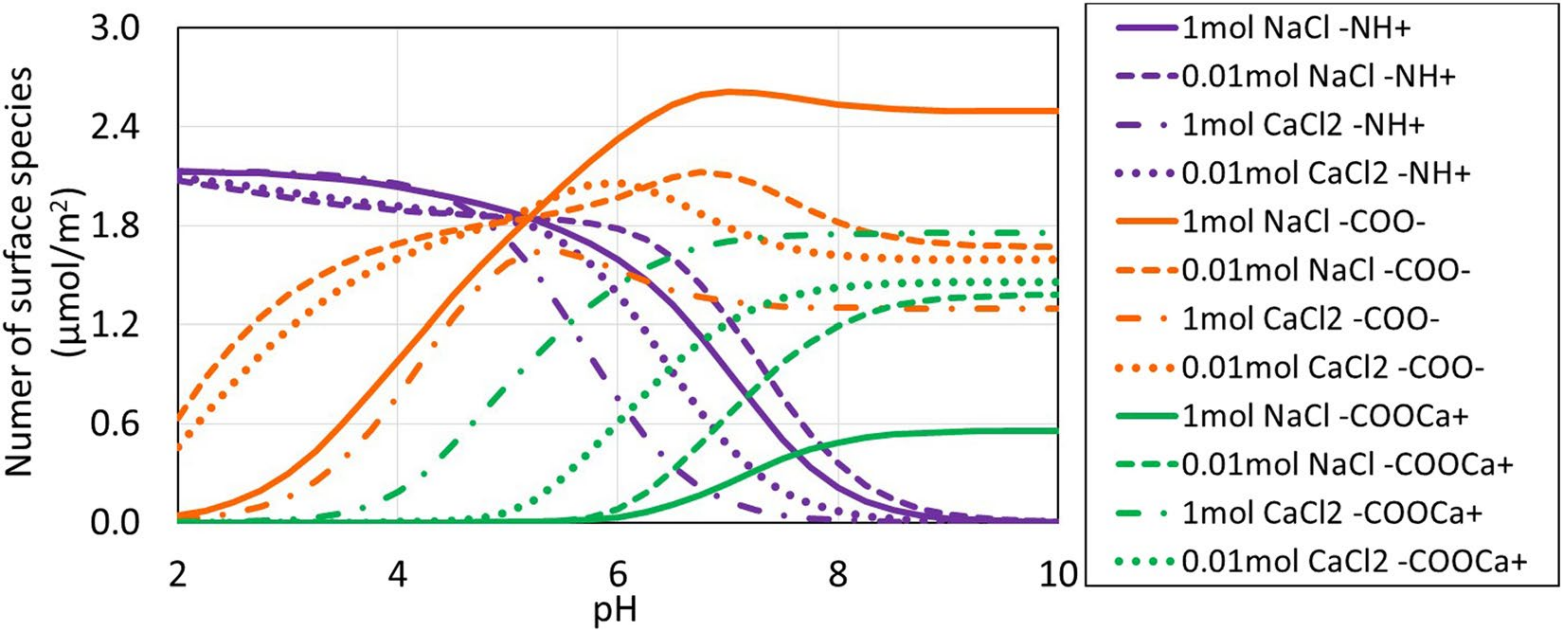
pH-dependent oil surface speciation in non-carbonated brine.

**Figure 4:**
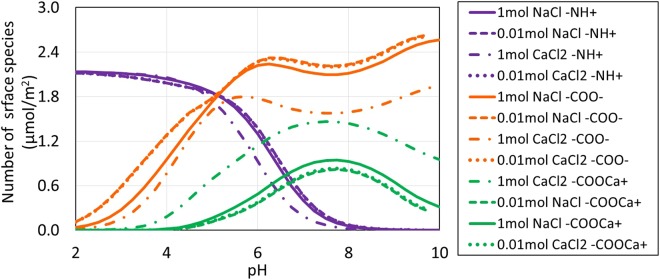
pH-dependent oil surface speciation in carbonated brine.

Figures 3 and 4 show calculated oil surface speciation in non-carbonated and carbonated NaCl and CaCl_2_ brines. Calculation results are listed in Tables 2 and 3 in Supplementary Information. The calculated amount of -NH^+^ decreases with increasing pH regardless of ion type and salinity for both non-carbonated and carbonated brines as pH controls the amount of-NH^+^ through Reaction 1 (Table 1) shifting to the left^25,29^. The calculated amount of -COO^−^ increases with increasing pH but decreases due to the formation of –COOCa^+^ for non-carbonated brines (Fig. 3). The same trend is observed in carbonated brines (Fig. 4), but with an increase of -COO^−^ with increasing pH due to the formation of CO_3_^2−^, which decreases Ca^2+^. Note: the amount of –COOCa^+^ depends on dissolved Ca levels and to a lesser extent ionic strength because of their effect on surface species concentrations and the Ca^2+^ activity coefficient^25^. Keeping in mind that the surface speciation responds to dissolved phase concentrations that, through calcite equilibria, are set by pH and amount of carbonation (*in situ* P_CO2_). For example, Ca^2+^ levels and ionic strength are higher at low pH and in carbonated brine. The PHREEQC surface complexation calculation tracks each of the competing factors while maintaining equilibrium with calcite.

### Speciation of Calcite/Brine Interfaces

**Figure 5:**
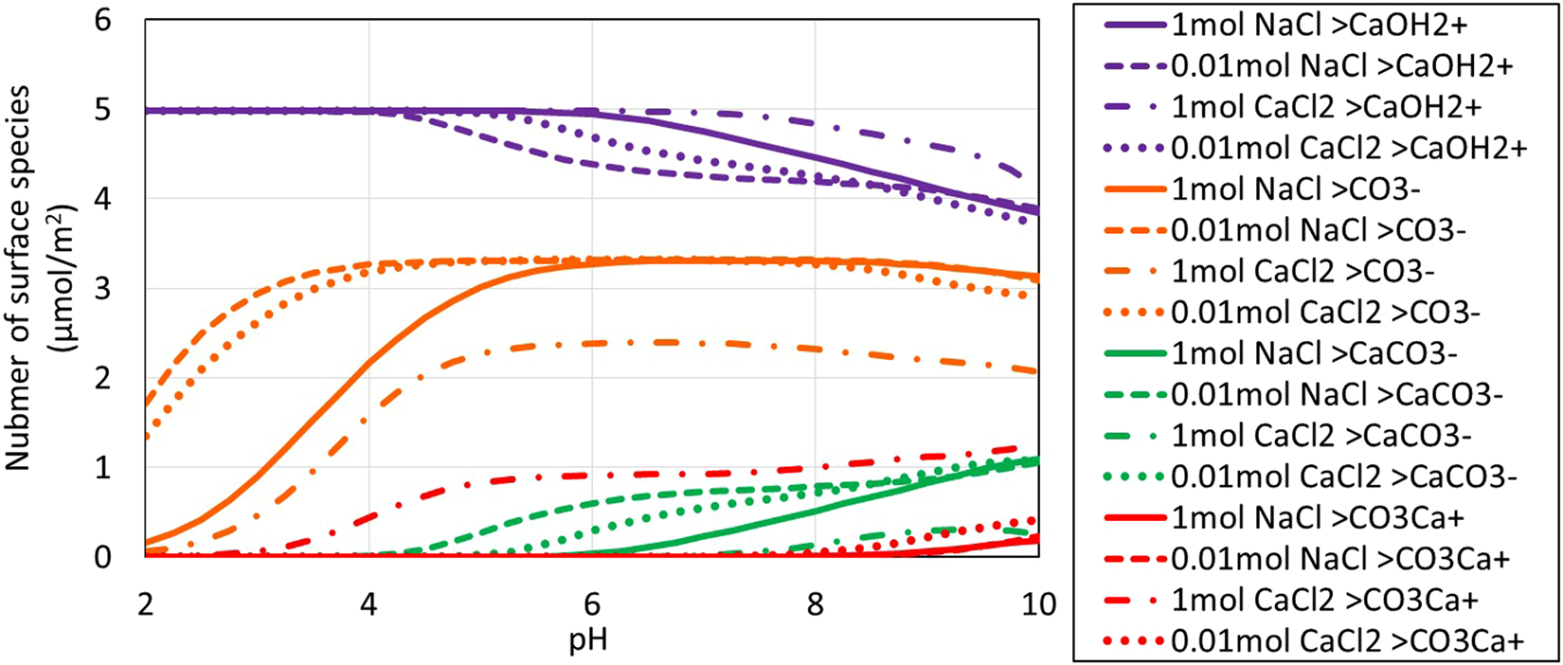
pH-dependent calcite surface speciation in non-carbonated brine.

**Figure 6:**
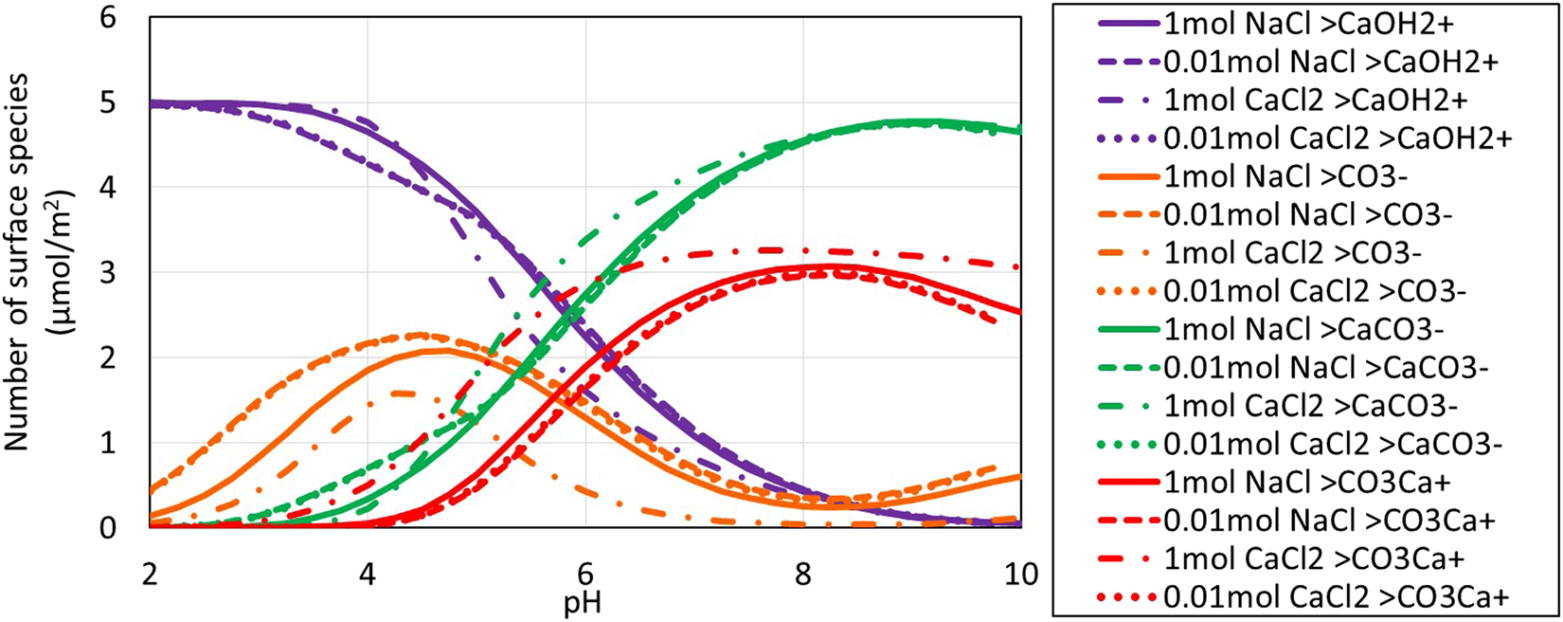
pH-dependent calcite surface speciation in carbonated brine.

**Figure 7:**
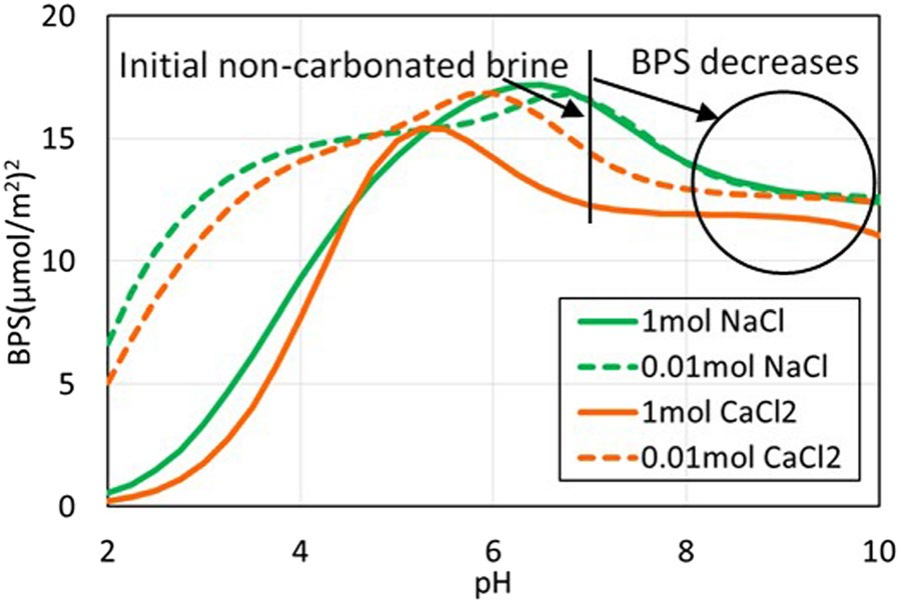
Bond Product Sum vs. pH in non-carbonated brine. The PHREEQC calculated pH of non-carbonated brine with calcite in equilibrium for 1 mol/L NaCl, CaCl_2_, and 0.01 mol/L NaCl, CaCl_2_, were 9.8, 8.2, 9.0, and 9.9 at P_CO2_ = 0 psi, and 25 °C. The initial pH of all fluids before equilibration with calcite was 7.

**Figure 8:**
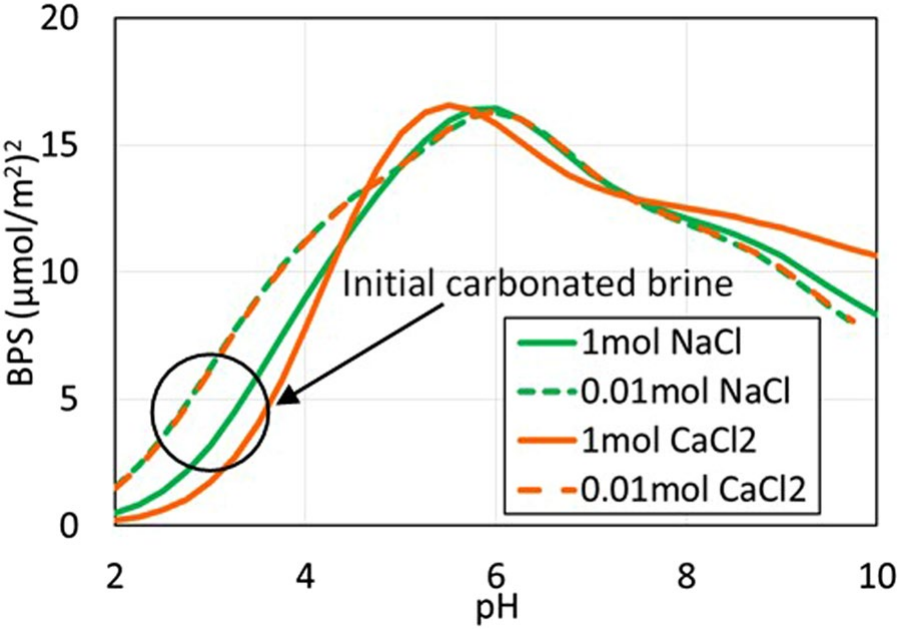
Bond Product Sum vs. pH for carbonated brine. The PHREEQC calculated pH of carbonated brine for 1 mol/L NaCl, CaCl_2_, and 0.01 mol/L NaCl, CaCl_2_, were 4.9, 4.1, 4.9, and 4.8 at P_CO2_ = 3000 psi, and 25 °C after equilibration with calcite. The calculated pre-calcite equilibration brine pH was 3.0, 2.6, 3.0, and 3.0 for 1 mol/L NaCl, CaCl_2_, and 0.01 mol/L NaCl, CaCl_2_, respectively, at P_CO2_ = 3000 psi, and 25 °C.

Figures 5 and 6 show calculated calcite surface speciation in non-carbonated and carbonated NaCl and CaCl_2_ brines. Calculation results are listed in Tables 4 and 5 in Supplementary Information. Note that the legends in Figs 5–8 refer to initial solution compositions. Final solution compositions are influenced by calcite dissolution and P_CO2_. Because in the CaCO_3_-H_2_O-CO_2_ system CO_2_, pH, and Ca^2+^ are coupled, ionic strength is particularly sensitive to pH and P_CO2_-dependent calcite dissolution reactions. So calcite dissolution in the pH < 4 for example causes calculated ionic strengths to be well above 1 M.

In both non-carbonated and carbonated solutions, low pH calcite surface charge is dominated by >CaOH_2_^+^. >CaOH_2_^+^ is the most abundant surface species at high pH as well in non-carbonated solutions. Increasing pH favors a decrease in >CaOH_2_^+^ and an increase in >CaCO_3_^−^, >CO_3_^−^, and >CO_3_Ca^+^ for a given available Ca^+2^. In carbonated solutions, high pHs and bicarbonate prompt appreciable formation of >CaCO_3_^−^, >CO_3_^−^, and >CO_3_Ca^+^.

Two shifts that stand out between the non-carbonated and carbonated cases are the conversion of >CaOH_2_^+^ to >CaCO_3_^−^ and >CO_3_^−^ to >CO_3_Ca^+^ with increasing CO_2_. These reactions are driven by respectively the higher bicarbonate and calcium levels in CO_2_-charged brine:

$$\begin{array}{c} > {{\rm{CaOH}}}_{2}^{+}+{{\rm{HCO}}}_{3}^{-}\leftrightarrow  > \,{{\rm{CaCO}}}_{3}^{-}+{{\rm{H}}}_{2}{\rm{O}}\\  > {{\rm{CO}}}_{3}^{-}+{{\rm{Ca}}}^{+2}\leftrightarrow  > \,{{\rm{CO}}}_{3}{{\rm{Ca}}}^{+}\end{array}$$

### Calculation of Oil-on-Calcite Wetting

We combined the calculated oil and calcite speciation above into a bond product sum, BPS, the number of electrostatic bridges between the oil and calcite. Again, the BPS is a measure of electrostatic attraction between oil and calcite, is proportional to measured contact angles^21,30^, and is therefore a useful predictor of wetting. For our system, the bond product sum is the total of six concentration products that quantify the strength of six electrostatic bridges between oppositely charged oil and calcite species, as noted above. For natural systems containing sulphate the BPS would also include, for example, a [>CaSO_4_^−^][-NH^+^] term.

Figures 7 and 8 show the bond product sum for non-carbonated and carbonated conditions. Calculation outputs are listed in Table 6 and 7 of Supplementary Information. The most important electrostatic bridges are [>CaOH_2_^+^][-COO^−^], [>CO_3_^−^][-COOCa^+^], and [>CaCO_3_^−^][-COOCa^+^]; the first of these bridges provides most of the oil-calcite electrostatic linking.

The pH in non-carbonated brines increased from 7 to 10 after equilibration with calcite which decreases the BPS (Fig. 7) and the contact angle (Fig. 1). In contrast, in carbonated brines, the pH decreases to below 4 which decreases the BPS to almost three times than the non-carbonated brine (Fig. 8), accounting for the contact angle decrease in a range of 20 to 80° with various brines (Fig. 1) thus more hydrophilicity system. The pH difference between calcite-equilibrated carbonated and non-carbonated brine largely accounts for why Teklu *et al*.^10^ and we observed a dramatic contact angle decrease in carbonated brine (Fig. 1). Specifically, electrostatic adhesion decreases with carbonation because of a decrease in pH. In a carbonate reservoir the reduction in electrostatic adhesion with carbonation ultimately means greater oil recovery because it causes an increase in oil relative permeability^31^.

Although BPS prediction appears to be in line with contact angle measurements on calcite surfaces, to complement the BPS estimates and provide deeper thermodynamic insights to the nature the physics which controls wettability of brine-oil-carbonate, we computed surface potential of brine-oil and brine-calcite in light of diffuse double layer to calculate total disjoining pressure of oil-brine-carbonate system in non-carbonated and carbonate brine within DLVO framework^32^ as the sum

1 $${{\rm{\Pi }}}_{Total}={{\rm{\Pi }}}_{electrical}-H/6\pi {L}^{3}$$

where **II**_*Total*_ is the disjoining pressure of the specific intermolecular interactions which reflects the interactive forces between the interfaces of brine-oil and brine-rock. **II**_*electrical*_ is the electrostatic forces due to the development of the charges between interacting surfaces. A brief introduction of the forces and calculation procedures were presented elsewhere^33^. The Hamaker constant for oil-brine-rock in water is approximately 1 × 10^−20^ J^34^. Melrose^35^ used Hamaker constants ranging from 0.3 to 0.9 × 10^−20^ J. In this study, 0.81 × 10^−20^ J was used as the Hamaker constant^34^. We did not consider the structural forces to model the total disjoining pressure due to the fact that the structural forces are short-range interactions over a distance of less than 5 nm compared with long-range interactions^36^, e.g., London-van der Waals and electrical double layer forces.

We computed the brine chemistry and surface potential of fluid-fluid and fluid-rock with considering calcite dissolution and water uptake of CO_2_ for carbonate brine using PHREEQC, as shown in Table 9 and Table 10 listed in Supplementary Information. Constant charge (CC) and constant potential (CP) conditions based on the linear P-B expression^37^ were used to compute total disjoining pressure versus interfacial separation of the oil and calcite surfaces. The two conditions represent upper and lower bounding curves on the total disjoining pressure^25,37^.

**Figure 9:**
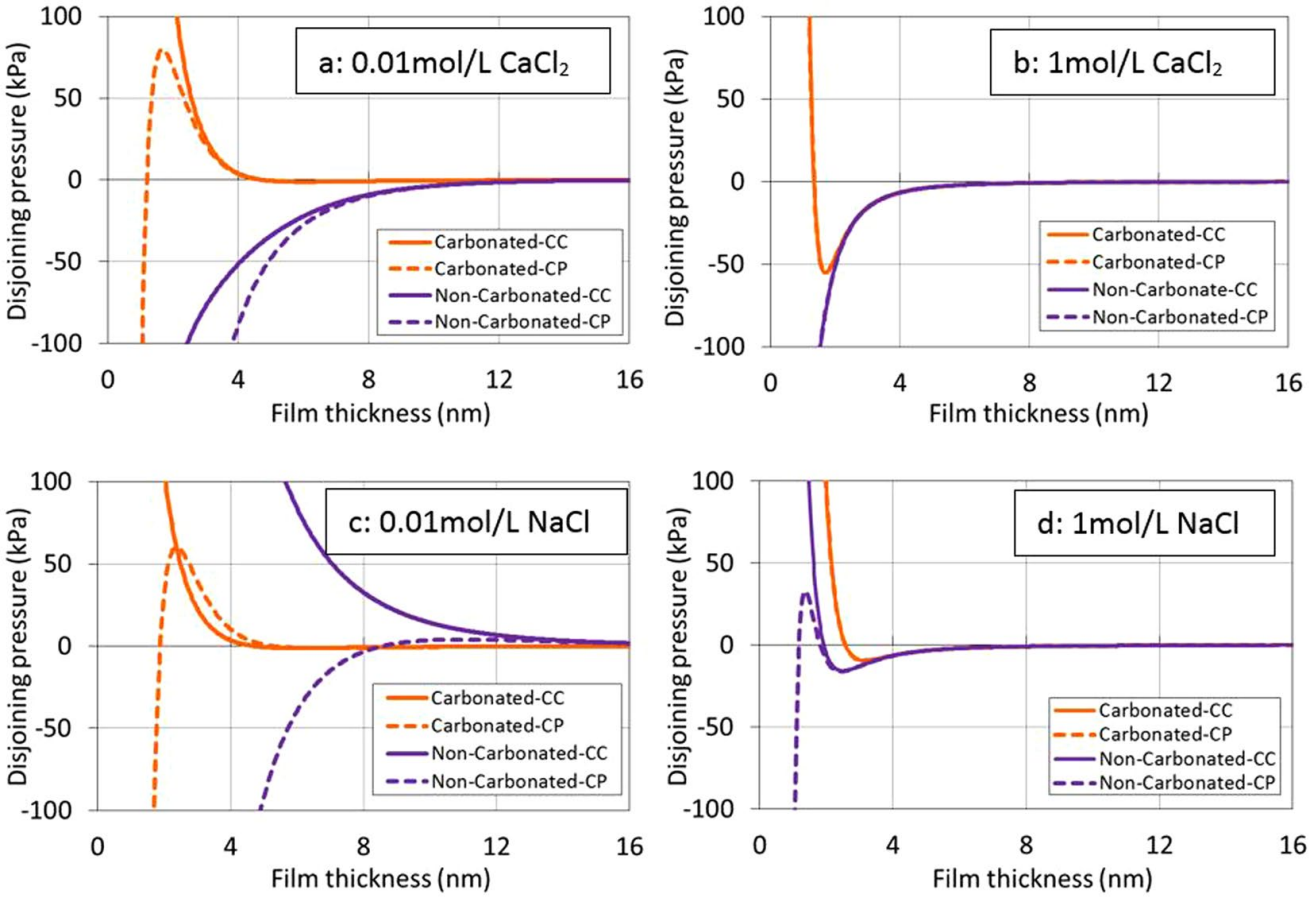
Total disjoing pressure under the condition of constant charge (solid lines) and constant potential (dotted lines) versus film thickness in the presence of carbonated and non-carbonated brines with different ion type and salinity. (CC means constant charges condition, and CP represents constant potential condition).

Figure 9 shows the isotherms of total disjoining pressure versus separation distance between oil and calcite surfaces across various brines. Positive pressure indicates repulsion, and negative pressure implies attraction. In the presence of 0.01 mol/L CaCl_2_ and NaCl solution, carbonated brine gave a positive disjoining pressure which exhibits a progressively more repulsive barrier on approach, implying a strongly water-wet system in line with contact angle measurements. Non-carbonated brine gave a negative disjoining pressure indicates an oil-wet system except 0.01 mol/L NaCl at constant charge (CC). This however contradicts contact angle results, which show a water-wet system although contact angle is 20 to 30° more than the contact angle in carbonated brines. This is because non-carbonated brine gives a strongly negative surface potential, whereas the surface potential of brine-calcite remains positive, triggering attractive forces. We believe that both electrostatic and nonelectrostatic physisorption together with competitive ion chemisorption (ion exchange and surface complexation modelling)^38^ would be combined to better account for the total disjoining pressure.

In the presence of 1 mol/L CaCl_2_ and NaCl solution, carbonated brine yields a relatively lower adhesion compared to non-carbonated brine, indicating a lower contact angle in carbonated brine in line with experiments. However, the same contradiction remains, showing that both carbonated and non-carbonated brine gave negative disjoining pressure, signifying an oil-wet system. We believe that double layer collapse may be one of the main reasons to account for this negative disjoining pressure due to the high ionic strength^39^. Together, although the disjoining pressure does not completely predict the contact angle results, DLVO and surface complexation modelling predict the same trend over the contact angle results, showing that carbonated brine leads to less adhesion compared to non-carbonated brine thus hydrophilicity in line with contact angle results. In addition, our surface potential results also show that carbonation plays a minor role in surface potential of brine-calcite in the presence of CaCl_2_ (Table 10 in Supplementary Information), confirming that Ca level likely dominates brine-calcite surface potential thus zeta potential rather than pH in line with Mahrouqi *et al*.^39^.

## Implications and Conclusions

To better predict and manage CO_2_ geological storage and enhanced oil recovery in carbonate oil reservoirs, we aimed to understand oil-CO_2_-brine-carbonate system wettability by measuring oil contact angle in carbonated and non-carbonated brines. We also coupled surface complexation/CO_2_ and calcite dissolution model, and accurately predicted measured oil-on-calcite contact angles in NaCl and CaCl_2_ solutions with and without CO_2_. To further complements surface complexation modelling, DLVO theory was used to calculate disjoining pressure at constant charge and constant potential conditions, confirming that DLVO and surface complexation modelling predict the same trend. Contact angle results show that carbonated water increases hydrophilicity. Reduced salinity increased hydrophilicity as did Ca^2+^. Our coupled surface complexation/CO_2_ and mineral dissolution model provides a mechanistic rationale for the CO_2_-induced wettability shift, and a means for coupling such observations into larger reservoir simulators. The latter might provide a path for more effectively tuning CO_2_ EOR to increase oil recovery from carbonate reservoirs. That being said, uncertainties remain. The surface complexation modelling might be improved by developing a more precise picture of the oil-water interface chemistry, specifically by verifying more closely the identities and surface acidity constants of surface polar groups e.g. through zeta potential measurements. The impact of salinity on oil and calcite surface complexation in high TDS solutions must be verified. Alternative calcite surface complexation stoichiometries than those in Table 1 exist^27^. Our preliminary calculations using the calcite surface stoichiometries of Song *et al*.^27^ predict the same trends seen above although the absolute values of the calculated BPS are different (Fig. 9 and Table 8 in Supplementary Information).

## Methods

### Substrates

Calcite minerals supplied by Ward’s Science were used in the contact angle tests. X-Ray Diffraction (XRD) tests confirmed that the composition of substrates were 100% calcite. To avoid any hysteresis and contamination the natural mineral surfaces (cleavage) were used as pendent spots.

Prior to experiments, substrates were cleaned with solvents (e.g., toluene and methanol) to remove any traces of organic and inorganic contaminants. Substrates were then rinsed with equilibrated deionised water to prevent undesired dissolution and dried in an oven at moderate temperature of 60 °C. Then, clean and dry substrates were exposed to air plasma for 10 min to remove organic surface contamination^40^. We also imaged the cleaved calcite substrate to obtain the surface roughnesusing atomic force microscopy (AFM) (WITec, ALPHA 300 RA for combined Raman-AFM imaging). Results show that the surface roughness was in a range of 0 to 4.8 nm^30^, implying that the surface roughness effect on contact angle should be negligible^16,41^.

### Liquids Preparation

Texas crude oil from the United States was used in contact angle tests. Chemical analysis of crude oil indicated the acid and base number were 1.7 and 1.2 mg KOH/g, respectively. To prepare carbonated brines, 1.0 mole and 0.01 mole of NaCl and CaCl_2_ brines were prepared and individually loaded in a reactor. CO_2_ gas was injected in the reactor through a syringe pump with the aid of a compressor and mixed with the brine at 3000 psi and 25 °C until the brine was saturated with CO_2_ gas. Saturated brine was transferred into an accumulator and maintained under pressure until the experiment was carried out.

### Experimental Procedure

Contact angle experiments were measured using a Vinci IFT apparatus (see Fig. 1 in Xie *et al*.^28^). All contact angles were measured at 3000 psi and 25 °C conditions. Calcite substrates were mounted on the apparatus turn table and placed inside the high pressure high temperature (HPHT) cell and sealed and vacuumed until state of vacuum was attained. The pressure cell was then filled with the desired brine and pressurised to 3000 psi. Subsequently, the experimental oil was slowly and steadily introduced into the cell through a capillary needle (0.64 mm diameter) until a droplet was formed. The droplet was then released on the substrate, and integrated software was utilised to measure left and right contact angles between substrate and the oil droplet. Contact angles were continuously recorded until equilibrium was achieved where contact angle became stable. This process was repeated for CO_2_-saturated brines. Throughout the experiment test pressure was closely monitored and maintained to prevent depressurisation of cell, and desaturation of the brine.

### Simulation Methods

Surface complexation modelling (and DLVO theory) presumes an electrical double layer at each interface and the existence of charged surface species whose concentrations depend upon the chemical makeup of the water and the oil and mineral surface^28^. Surface equilibria and constants^23,42–44^ are listed in Table 1. The surface species concentrations were calculated using PHREEQC version 3.3.9 (Parkhurst and Appelo 2013) and a diffuse layer surface model. The calcite surface site density was assumed to be 5 sites/nm^2,22^. The oil/calcite surface area was set to 0.11 m^2^/g^22^.

## Electronic supplementary material

Supplementary Information
