# Supplementary material for: Electrostatic Origins of CO2-Increased Hydrophilicity in Carbonate Reservoirs
Source: Sci Rep. 2018 Dec 6;8:17691. doi: 10.1038/s41598-018-35878-3 (PMC6283841; doi:10.1038/s41598-018-35878-3)
Supplement: Supplementary file 1 — Supplementary Information [file 41598_2018_35878_MOESM1_ESM.docx]

Electrostatic Origins of CO_2_-Increased Hydrophilicity in Carbonate Reservoirs

Yongqiang Chen ^a^, Ahmad Sari ^a^, Quan Xie ^a^*, Patrick V. Brady ^b^, Md Mofazzal Hossain ^a^, Ali Saeedi ^a^

^a^Department of Petroleum Engineering, Curtin University, 26 Dick Perry Avenue, 6151 Kensington, Western Australia, Australia

^b^Sandia National Laboratories, Albuquerque, New Mexico 87185-0754, United States

Figure 9 shows the BPS in the carbonated brines using Song et al.’s ^27^ model with CO_2_ and mineral dissolution using alternative calcite surface stoichiometries. Song et al.’s model predicts the same trend as our model, showing that carbonated brines gave a much lower BPS compared to in-situ reservoir condition (pH 6-7) although the absolute value is different.


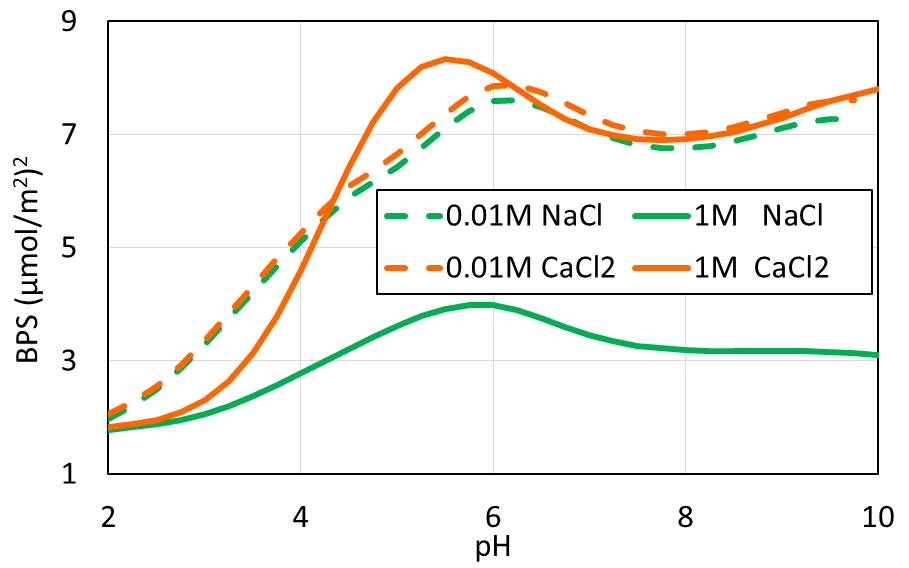


Initial carbonated brine

Figure 9. Bond Product Sum for the carbonated system using Song et al.’s ^27^ model with CO_2_ and mineral dissolution using alternative calcite surface stoichiometries.

Table 2. pH-dependent oil surface speciation in non-carbonated brine.

| Oil surface species in non-carbonated brine for Figure 3 in article file | | | | | | | | | | | | |
| --- | --- | --- | --- | --- | --- | --- | --- | --- | --- | --- | --- | --- |
| pH | –NH^+^ (µmol/m^2^) | | | | –COO^-^ (µmol/m^2^) | | | | –COOCa^+^ (µmol/m^2^) | | | |
|  | CaCl_2_ | CaCl_2_ | NaCl | NaCl | CaCl_2_ | CaCl_2_ | NaCl | NaCl | CaCl_2_ | CaCl_2_ | NaCl | NaCl |
|  | 1M | 0.01M | 1M | 0.01M | 1M | 0.01M | 1M | 0.01M | 1M | 0.01M | 1M | 0.01M |
| 2.00 | 2.13 | 2.09 | 2.13 | 2.08 | 0.02 | 0.45 | 0.04 | 0.63 | 0.00 | 0.00 | 0.00 | 0.00 |
| 2.25 | 2.13 | 2.07 | 2.13 | 2.05 | 0.03 | 0.65 | 0.07 | 0.87 | 0.00 | 0.00 | 0.00 | 0.00 |
| 2.50 | 2.13 | 2.05 | 2.12 | 2.02 | 0.05 | 0.84 | 0.12 | 1.08 | 0.01 | 0.00 | 0.00 | 0.00 |
| 2.75 | 2.12 | 2.03 | 2.12 | 1.99 | 0.09 | 1.01 | 0.19 | 1.25 | 0.01 | 0.00 | 0.00 | 0.00 |
| 3.00 | 2.12 | 2.01 | 2.11 | 1.97 | 0.15 | 1.17 | 0.30 | 1.38 | 0.02 | 0.00 | 0.00 | 0.00 |
| 3.25 | 2.11 | 1.98 | 2.10 | 1.95 | 0.25 | 1.31 | 0.43 | 1.48 | 0.03 | 0.00 | 0.00 | 0.00 |
| 3.50 | 2.10 | 1.96 | 2.08 | 1.93 | 0.38 | 1.42 | 0.60 | 1.57 | 0.06 | 0.00 | 0.00 | 0.00 |
| 3.75 | 2.08 | 1.94 | 2.06 | 1.91 | 0.56 | 1.52 | 0.79 | 1.64 | 0.10 | 0.00 | 0.00 | 0.00 |
| 4.00 | 2.05 | 1.92 | 2.03 | 1.89 | 0.77 | 1.60 | 0.98 | 1.69 | 0.18 | 0.00 | 0.00 | 0.00 |
| 4.25 | 2.01 | 1.90 | 2.00 | 1.88 | 1.00 | 1.67 | 1.18 | 1.73 | 0.31 | 0.00 | 0.00 | 0.00 |
| 4.50 | 1.95 | 1.88 | 1.97 | 1.87 | 1.24 | 1.73 | 1.37 | 1.77 | 0.47 | 0.01 | 0.00 | 0.00 |
| 4.75 | 1.85 | 1.86 | 1.93 | 1.86 | 1.44 | 1.78 | 1.55 | 1.80 | 0.65 | 0.02 | 0.00 | 0.00 |
| 5.00 | 1.71 | 1.83 | 1.88 | 1.85 | 1.57 | 1.84 | 1.72 | 1.83 | 0.84 | 0.06 | 0.00 | 0.00 |
| 5.25 | 1.52 | 1.79 | 1.83 | 1.84 | 1.63 | 1.92 | 1.89 | 1.86 | 1.01 | 0.14 | 0.00 | 0.00 |
| 5.50 | 1.28 | 1.70 | 1.77 | 1.83 | 1.64 | 1.99 | 2.04 | 1.88 | 1.17 | 0.27 | 0.01 | 0.01 |
| 5.75 | 1.01 | 1.57 | 1.69 | 1.81 | 1.59 | 2.05 | 2.19 | 1.92 | 1.31 | 0.43 | 0.02 | 0.03 |
| 6.00 | 0.75 | 1.38 | 1.59 | 1.78 | 1.53 | 2.06 | 2.33 | 1.97 | 1.44 | 0.61 | 0.03 | 0.08 |
| 6.25 | 0.52 | 1.16 | 1.47 | 1.71 | 1.46 | 2.02 | 2.44 | 2.04 | 1.54 | 0.78 | 0.06 | 0.18 |
| 6.50 | 0.34 | 0.91 | 1.32 | 1.61 | 1.41 | 1.96 | 2.53 | 2.09 | 1.62 | 0.95 | 0.11 | 0.32 |
| 6.75 | 0.21 | 0.67 | 1.13 | 1.44 | 1.37 | 1.87 | 2.59 | 2.12 | 1.67 | 1.09 | 0.17 | 0.48 |
| 7.00 | 0.13 | 0.47 | 0.92 | 1.24 | 1.34 | 1.79 | 2.61 | 2.11 | 1.70 | 1.21 | 0.24 | 0.65 |
| 7.25 | 0.07 | 0.30 | 0.70 | 1.00 | 1.32 | 1.72 | 2.61 | 2.05 | 1.73 | 1.30 | 0.31 | 0.82 |
| 7.50 | 0.04 | 0.19 | 0.50 | 0.76 | 1.31 | 1.67 | 2.59 | 1.97 | 1.74 | 1.36 | 0.38 | 0.97 |
| 7.75 | 0.02 | 0.11 | 0.33 | 0.54 | 1.30 | 1.64 | 2.56 | 1.89 | 1.74 | 1.40 | 0.44 | 1.10 |
| 8.00 | 0.01 | 0.07 | 0.21 | 0.36 | 1.30 | 1.62 | 2.54 | 1.82 | 1.75 | 1.42 | 0.49 | 1.19 |
| 8.25 | 0.01 | 0.04 | 0.13 | 0.23 | 1.30 | 1.61 | 2.52 | 1.76 | 1.75 | 1.44 | 0.52 | 1.26 |
| 8.50 | 0.00 | 0.02 | 0.08 | 0.14 | 1.30 | 1.60 | 2.51 | 1.73 | 1.75 | 1.45 | 0.53 | 1.31 |
| 8.75 | 0.00 | 0.01 | 0.04 | 0.08 | 1.30 | 1.60 | 2.50 | 1.70 | 1.75 | 1.45 | 0.54 | 1.34 |
| 9.00 | 0.00 | 0.01 | 0.02 | 0.05 | 1.30 | 1.59 | 2.50 | 1.69 | 1.75 | 1.46 | 0.55 | 1.36 |
| 9.25 | 0.00 | 0.00 | 0.01 | 0.03 | 1.30 | 1.59 | 2.49 | 1.68 | 1.75 | 1.46 | 0.55 | 1.37 |
| 9.50 | 0.00 | 0.00 | 0.01 | 0.02 | 1.30 | 1.59 | 2.49 | 1.68 | 1.75 | 1.46 | 0.56 | 1.37 |
| 9.75 | 0.00 | 0.00 | 0.00 | 0.01 | 1.30 | 1.59 | 2.49 | 1.67 | 1.75 | 1.46 | 0.56 | 1.38 |
| 10.00 | 0.00 | 0.00 | 0.00 | 0.00 | 1.30 | 1.59 | 2.49 | 1.67 | 1.75 | 1.46 | 0.56 | 1.38 |

Table 3. pH-dependent oil surface speciation in carbonated brine.

| Oil surface species in carbonated brine for Figure 4 in article file | | | | | | | | | | | | |
| --- | --- | --- | --- | --- | --- | --- | --- | --- | --- | --- | --- | --- |
| pH | –NH^+^ (µmol/m^2^) | | | | –COO^-^ (µmol/m^2^) | | | | –COOCa^+^ (µmol/m^2^) | | | |
|  | CaCl_2_ | CaCl_2_ | NaCl | NaCl | CaCl_2_ | CaCl_2_ | NaCl | NaCl | CaCl_2_ | CaCl_2_ | NaCl | NaCl |
|  | 1M | 0.01M | 1M | 0.01M | 1M | 0.01M | 1M | 0.01M | 1M | 0.01M | 1M | 0.01M |
| 2.00 | 2.13 | 2.12 | 2.13 | 2.12 | 0.02 | 0.11 | 0.04 | 0.12 | 0.00 | 0.00 | 0.00 | 0.00 |
| 2.25 | 2.13 | 2.11 | 2.13 | 2.11 | 0.03 | 0.19 | 0.06 | 0.19 | 0.00 | 0.00 | 0.00 | 0.00 |
| 2.50 | 2.12 | 2.10 | 2.13 | 2.10 | 0.05 | 0.29 | 0.11 | 0.29 | 0.01 | 0.00 | 0.00 | 0.00 |
| 2.75 | 2.12 | 2.09 | 2.12 | 2.09 | 0.09 | 0.42 | 0.18 | 0.43 | 0.01 | 0.00 | 0.00 | 0.00 |
| 3.00 | 2.12 | 2.08 | 2.11 | 2.08 | 0.15 | 0.58 | 0.27 | 0.59 | 0.02 | 0.00 | 0.00 | 0.00 |
| 3.25 | 2.11 | 2.06 | 2.10 | 2.06 | 0.24 | 0.76 | 0.41 | 0.76 | 0.03 | 0.00 | 0.00 | 0.00 |
| 3.50 | 2.10 | 2.04 | 2.09 | 2.04 | 0.37 | 0.93 | 0.57 | 0.94 | 0.06 | 0.00 | 0.00 | 0.00 |
| 3.75 | 2.08 | 2.01 | 2.07 | 2.01 | 0.55 | 1.11 | 0.76 | 1.12 | 0.11 | 0.00 | 0.01 | 0.00 |
| 4.00 | 2.06 | 1.98 | 2.04 | 1.98 | 0.77 | 1.28 | 0.96 | 1.28 | 0.18 | 0.01 | 0.02 | 0.01 |
| 4.25 | 2.02 | 1.95 | 2.01 | 1.95 | 1.00 | 1.44 | 1.16 | 1.44 | 0.29 | 0.02 | 0.04 | 0.02 |
| 4.50 | 1.96 | 1.92 | 1.97 | 1.92 | 1.24 | 1.58 | 1.36 | 1.58 | 0.42 | 0.04 | 0.06 | 0.04 |
| 4.75 | 1.87 | 1.89 | 1.92 | 1.89 | 1.45 | 1.67 | 1.55 | 1.68 | 0.56 | 0.08 | 0.11 | 0.07 |
| 5.00 | 1.75 | 1.85 | 1.86 | 1.85 | 1.62 | 1.78 | 1.73 | 1.78 | 0.69 | 0.12 | 0.17 | 0.12 |
| 5.25 | 1.60 | 1.78 | 1.77 | 1.79 | 1.73 | 1.91 | 1.90 | 1.91 | 0.81 | 0.17 | 0.23 | 0.16 |
| 5.50 | 1.40 | 1.69 | 1.66 | 1.69 | 1.79 | 2.05 | 2.04 | 2.05 | 0.92 | 0.23 | 0.31 | 0.22 |
| 5.75 | 1.18 | 1.55 | 1.50 | 1.56 | 1.80 | 2.18 | 2.16 | 2.18 | 1.03 | 0.30 | 0.39 | 0.28 |
| 6.00 | 0.93 | 1.37 | 1.30 | 1.38 | 1.78 | 2.27 | 2.22 | 2.28 | 1.14 | 0.38 | 0.49 | 0.36 |
| 6.25 | 0.68 | 1.15 | 1.07 | 1.16 | 1.73 | 2.31 | 2.24 | 2.32 | 1.24 | 0.48 | 0.60 | 0.46 |
| 6.50 | 0.47 | 0.91 | 0.82 | 0.92 | 1.68 | 2.31 | 2.22 | 2.32 | 1.33 | 0.57 | 0.70 | 0.56 |
| 6.75 | 0.31 | 0.67 | 0.59 | 0.68 | 1.64 | 2.28 | 2.18 | 2.30 | 1.39 | 0.67 | 0.79 | 0.65 |
| 7.00 | 0.19 | 0.46 | 0.40 | 0.47 | 1.60 | 2.25 | 2.14 | 2.26 | 1.43 | 0.74 | 0.86 | 0.73 |
| 7.25 | 0.11 | 0.30 | 0.26 | 0.31 | 1.59 | 2.22 | 2.11 | 2.23 | 1.45 | 0.80 | 0.91 | 0.78 |
| 7.50 | 0.07 | 0.19 | 0.16 | 0.20 | 1.58 | 2.20 | 2.10 | 2.22 | 1.46 | 0.83 | 0.94 | 0.81 |
| 7.75 | 0.04 | 0.12 | 0.10 | 0.12 | 1.58 | 2.20 | 2.10 | 2.22 | 1.46 | 0.84 | 0.94 | 0.82 |
| 8.00 | 0.02 | 0.07 | 0.06 | 0.07 | 1.59 | 2.22 | 2.12 | 2.24 | 1.44 | 0.82 | 0.93 | 0.80 |
| 8.25 | 0.01 | 0.04 | 0.04 | 0.05 | 1.62 | 2.26 | 2.15 | 2.27 | 1.40 | 0.78 | 0.89 | 0.76 |
| 8.50 | 0.01 | 0.03 | 0.02 | 0.03 | 1.65 | 2.31 | 2.21 | 2.33 | 1.36 | 0.72 | 0.83 | 0.70 |
| 8.75 | 0.01 | 0.02 | 0.01 | 0.02 | 1.70 | 2.38 | 2.27 | 2.40 | 1.30 | 0.63 | 0.75 | 0.62 |
| 9.00 | 0.00 | 0.01 | 0.01 | 0.01 | 1.75 | 2.46 | 2.35 | 2.47 | 1.23 | 0.54 | 0.66 | 0.52 |
| 9.25 | 0.00 | 0.01 | 0.00 | 0.01 | 1.81 | 2.54 | 2.44 | 2.55 | 1.15 | 0.43 | 0.55 | 0.41 |
| 9.50 | 0.00 | 0.00 | 0.00 | 0.00 | 1.87 | 2.59 | 2.50 | 2.61 | 1.08 | 0.34 | 0.45 | 0.32 |
| 9.75 | 0.00 | 0.00 | 0.00 | 0.00 | 1.92 | 2.62 | 2.54 | 2.63 | 1.01 | 0.27 | 0.38 | 0.26 |
| 10.00 | 0.00 |  | 0.00 |  | 1.96 |  | 2.56 |  | 0.95 |  | 0.32 |  |

Table 4. pH-dependent calcite surface speciation in non-carbonated brine.

| Calcite surface speciation in non-carbonated brine (Figure 5, in article file) | | | | | | | | | | | | | | | | |
| --- | --- | --- | --- | --- | --- | --- | --- | --- | --- | --- | --- | --- | --- | --- | --- | --- |
| pH | >CaOH^2+^ (µmol/m^2^) | | | | >CO_3_^-^ (µmol/m^2^) | | | | >CaCO_3_^-^ (µmol/m^2^) | | | | >CO_3_Ca^+^ (µmol/m^2^) | | | |
|  | CaCl_2_ | CaCl_2_ | NaCl | NaCl | CaCl_2_ | CaCl_2_ | NaCl | NaCl | CaCl_2_ | CaCl_2_ | NaCl | NaCl | CaCl_2_ | CaCl_2_ | NaCl | NaCl |
|  | 1M | 0.01M | 1M | 0.01M | 1M | 0.01M | 1M | 0.01M | 1M | 0.01M | 1M | 0.01M | 1M | 0.01M | 1M | 0.01M |
| 2.00 | 4.98 | 4.98 | 4.98 | 4.98 | 0.06 | 1.35 | 0.16 | 1.71 | 0.00 | 0.00 | 0.00 | 0.00 | 0.01 | 0.00 | 0.00 | 0.00 |
| 2.25 | 4.98 | 4.98 | 4.98 | 4.98 | 0.11 | 1.74 | 0.27 | 2.15 | 0.00 | 0.00 | 0.00 | 0.00 | 0.01 | 0.00 | 0.00 | 0.00 |
| 2.50 | 4.98 | 4.98 | 4.98 | 4.98 | 0.18 | 2.09 | 0.42 | 2.49 | 0.00 | 0.00 | 0.00 | 0.00 | 0.02 | 0.00 | 0.00 | 0.00 |
| 2.75 | 4.98 | 4.98 | 4.98 | 4.98 | 0.29 | 2.39 | 0.63 | 2.75 | 0.00 | 0.00 | 0.00 | 0.00 | 0.04 | 0.00 | 0.00 | 0.00 |
| 3.00 | 4.98 | 4.98 | 4.98 | 4.98 | 0.46 | 2.63 | 0.90 | 2.94 | 0.00 | 0.00 | 0.00 | 0.00 | 0.07 | 0.00 | 0.00 | 0.00 |
| 3.25 | 4.98 | 4.98 | 4.98 | 4.98 | 0.69 | 2.83 | 1.21 | 3.08 | 0.00 | 0.00 | 0.00 | 0.00 | 0.12 | 0.00 | 0.00 | 0.00 |
| 3.50 | 4.98 | 4.98 | 4.98 | 4.98 | 0.97 | 2.99 | 1.53 | 3.17 | 0.00 | 0.00 | 0.00 | 0.00 | 0.19 | 0.00 | 0.00 | 0.00 |
| 3.75 | 4.98 | 4.98 | 4.98 | 4.98 | 1.28 | 3.10 | 1.85 | 3.23 | 0.00 | 0.00 | 0.00 | 0.01 | 0.30 | 0.00 | 0.00 | 0.00 |
| 4.00 | 4.98 | 4.98 | 4.98 | 4.97 | 1.58 | 3.18 | 2.16 | 3.26 | 0.00 | 0.00 | 0.00 | 0.02 | 0.44 | 0.00 | 0.00 | 0.00 |
| 4.25 | 4.98 | 4.98 | 4.98 | 4.94 | 1.84 | 3.24 | 2.43 | 3.29 | 0.00 | 0.00 | 0.00 | 0.04 | 0.57 | 0.00 | 0.00 | 0.00 |
| 4.50 | 4.98 | 4.98 | 4.98 | 4.89 | 2.04 | 3.27 | 2.67 | 3.30 | 0.00 | 0.00 | 0.00 | 0.09 | 0.69 | 0.00 | 0.00 | 0.00 |
| 4.75 | 4.98 | 4.97 | 4.98 | 4.81 | 2.18 | 3.29 | 2.86 | 3.30 | 0.00 | 0.01 | 0.00 | 0.18 | 0.78 | 0.00 | 0.00 | 0.00 |
| 5.00 | 4.98 | 4.96 | 4.98 | 4.71 | 2.27 | 3.30 | 3.01 | 3.31 | 0.00 | 0.02 | 0.00 | 0.28 | 0.83 | 0.00 | 0.00 | 0.00 |
| 5.25 | 4.98 | 4.92 | 4.98 | 4.61 | 2.33 | 3.31 | 3.12 | 3.31 | 0.00 | 0.06 | 0.00 | 0.37 | 0.87 | 0.00 | 0.00 | 0.00 |
| 5.50 | 4.98 | 4.86 | 4.97 | 4.52 | 2.36 | 3.31 | 3.20 | 3.31 | 0.00 | 0.12 | 0.01 | 0.46 | 0.89 | 0.00 | 0.00 | 0.00 |
| 5.75 | 4.98 | 4.78 | 4.96 | 4.44 | 2.38 | 3.32 | 3.24 | 3.31 | 0.00 | 0.20 | 0.02 | 0.54 | 0.90 | 0.00 | 0.00 | 0.00 |
| 6.00 | 4.98 | 4.69 | 4.94 | 4.38 | 2.39 | 3.32 | 3.27 | 3.31 | 0.00 | 0.29 | 0.04 | 0.60 | 0.91 | 0.00 | 0.00 | 0.00 |
| 6.25 | 4.98 | 4.61 | 4.91 | 4.34 | 2.39 | 3.32 | 3.29 | 3.31 | 0.00 | 0.37 | 0.07 | 0.64 | 0.92 | 0.00 | 0.00 | 0.00 |
| 6.50 | 4.97 | 4.54 | 4.87 | 4.30 | 2.39 | 3.32 | 3.30 | 3.32 | 0.01 | 0.44 | 0.11 | 0.68 | 0.92 | 0.00 | 0.00 | 0.00 |
| 6.75 | 4.97 | 4.48 | 4.81 | 4.27 | 2.39 | 3.32 | 3.31 | 3.32 | 0.01 | 0.50 | 0.17 | 0.71 | 0.93 | 0.00 | 0.00 | 0.00 |
| 7.00 | 4.96 | 4.43 | 4.75 | 4.25 | 2.39 | 3.31 | 3.31 | 3.32 | 0.02 | 0.55 | 0.23 | 0.73 | 0.93 | 0.01 | 0.00 | 0.00 |
| 7.25 | 4.94 | 4.39 | 4.68 | 4.24 | 2.38 | 3.31 | 3.31 | 3.32 | 0.04 | 0.59 | 0.30 | 0.75 | 0.94 | 0.01 | 0.00 | 0.00 |
| 7.50 | 4.92 | 4.34 | 4.61 | 4.22 | 2.37 | 3.30 | 3.31 | 3.32 | 0.06 | 0.63 | 0.37 | 0.76 | 0.95 | 0.02 | 0.01 | 0.00 |
| 7.75 | 4.88 | 4.30 | 4.54 | 4.20 | 2.35 | 3.29 | 3.31 | 3.32 | 0.09 | 0.67 | 0.44 | 0.77 | 0.98 | 0.03 | 0.01 | 0.00 |
| 8.00 | 4.84 | 4.26 | 4.46 | 4.19 | 2.32 | 3.27 | 3.31 | 3.31 | 0.14 | 0.72 | 0.52 | 0.79 | 1.00 | 0.05 | 0.01 | 0.01 |
| 8.25 | 4.78 | 4.21 | 4.39 | 4.18 | 2.29 | 3.25 | 3.30 | 3.31 | 0.19 | 0.76 | 0.59 | 0.80 | 1.03 | 0.07 | 0.02 | 0.01 |
| 8.50 | 4.72 | 4.15 | 4.31 | 4.16 | 2.26 | 3.21 | 3.29 | 3.30 | 0.23 | 0.82 | 0.67 | 0.82 | 1.06 | 0.11 | 0.03 | 0.02 |
| 8.75 | 4.67 | 4.08 | 4.23 | 4.13 | 2.23 | 3.16 | 3.28 | 3.29 | 0.27 | 0.88 | 0.75 | 0.84 | 1.09 | 0.16 | 0.05 | 0.03 |
| 9.00 | 4.61 | 4.01 | 4.15 | 4.11 | 2.21 | 3.10 | 3.25 | 3.27 | 0.30 | 0.94 | 0.83 | 0.87 | 1.12 | 0.22 | 0.07 | 0.05 |
| 9.25 | 4.55 | 3.93 | 4.06 | 4.07 | 2.18 | 3.04 | 3.23 | 3.24 | 0.31 | 1.00 | 0.91 | 0.90 | 1.14 | 0.28 | 0.09 | 0.08 |
| 9.50 | 4.46 | 3.86 | 3.98 | 4.01 | 2.16 | 2.99 | 3.20 | 3.20 | 0.31 | 1.05 | 0.98 | 0.95 | 1.17 | 0.33 | 0.13 | 0.12 |
| 9.75 | 4.32 | 3.79 | 3.91 | 3.95 | 2.12 | 2.94 | 3.16 | 3.15 | 0.29 | 1.08 | 1.05 | 1.00 | 1.20 | 0.38 | 0.16 | 0.18 |
| 10.00 | 4.12 | 3.72 | 3.84 | 3.89 | 2.06 | 2.91 | 3.13 | 3.09 | 0.26 | 1.08 | 1.10 | 1.06 | 1.26 | 0.41 | 0.19 | 0.23 |

Table 5. pH-dependent calcite surface speciation in carbonated brine.

| Calcite surface speciation in carbonated brine for Figure 6 in article file | | | | | | | | | | | | | | | | |
| --- | --- | --- | --- | --- | --- | --- | --- | --- | --- | --- | --- | --- | --- | --- | --- | --- |
| pH | >CaOH^2+^ (µmol/m^2^) | | | | >CO_3_^-^  (µmol/m^2^) | | | | >CaCO_3_^-^ (µmol/m^2^) | | | | >CO_3_Ca^+^ (µmol/m^2^) | | | |
|  | CaCl_2_ | CaCl_2_ | NaCl | NaCl | CaCl_2_ | CaCl_2_ | NaCl | NaCl | CaCl_2_ | CaCl_2_ | NaCl | NaCl | CaCl_2_ | CaCl_2_ | NaCl | NaCl |
|  | 1M | 0.01M | 1M | 0.01M | 1M | 0.01M | 1M | 0.01M | 1M | 0.01M | 1M | 0.01M | 1M | 0.01M | 1M | 0.01M |
| 2.00 | 4.97 | 4.97 | 4.99 | 4.97 | 0.06 | 0.43 | 0.15 | 0.44 | 0.00 | 0.00 | 0.00 | 0.01 | 0.01 | 0.00 | 0.00 | 0.00 |
| 2.25 | 4.97 | 4.96 | 4.99 | 4.96 | 0.10 | 0.65 | 0.24 | 0.67 | 0.00 | 0.01 | 0.00 | 0.01 | 0.01 | 0.00 | 0.00 | 0.00 |
| 2.50 | 4.97 | 4.94 | 4.99 | 4.94 | 0.17 | 0.91 | 0.38 | 0.93 | 0.00 | 0.03 | 0.00 | 0.03 | 0.02 | 0.00 | 0.00 | 0.00 |
| 2.75 | 4.97 | 4.90 | 4.98 | 4.90 | 0.28 | 1.20 | 0.58 | 1.22 | 0.00 | 0.07 | 0.01 | 0.07 | 0.04 | 0.00 | 0.00 | 0.00 |
| 3.00 | 4.97 | 4.84 | 4.97 | 4.83 | 0.45 | 1.47 | 0.83 | 1.49 | 0.01 | 0.14 | 0.02 | 0.14 | 0.07 | 0.00 | 0.00 | 0.00 |
| 3.25 | 4.96 | 4.73 | 4.94 | 4.72 | 0.67 | 1.71 | 1.11 | 1.73 | 0.02 | 0.24 | 0.05 | 0.25 | 0.13 | 0.00 | 0.01 | 0.00 |
| 3.50 | 4.94 | 4.60 | 4.88 | 4.59 | 0.93 | 1.91 | 1.39 | 1.92 | 0.04 | 0.38 | 0.11 | 0.39 | 0.21 | 0.01 | 0.01 | 0.01 |
| 3.75 | 4.88 | 4.45 | 4.79 | 4.44 | 1.21 | 2.06 | 1.65 | 2.06 | 0.10 | 0.53 | 0.21 | 0.54 | 0.34 | 0.01 | 0.03 | 0.01 |
| 4.00 | 4.76 | 4.29 | 4.65 | 4.28 | 1.45 | 2.16 | 1.85 | 2.17 | 0.23 | 0.69 | 0.34 | 0.70 | 0.51 | 0.03 | 0.06 | 0.03 |
| 4.25 | 4.52 | 4.13 | 4.48 | 4.12 | 1.58 | 2.23 | 2.00 | 2.24 | 0.47 | 0.85 | 0.52 | 0.86 | 0.75 | 0.07 | 0.11 | 0.06 |
| 4.50 | 4.16 | 3.96 | 4.27 | 3.96 | 1.57 | 2.26 | 2.07 | 2.27 | 0.83 | 1.03 | 0.73 | 1.03 | 1.05 | 0.15 | 0.22 | 0.14 |
| 4.75 | 3.69 | 3.81 | 4.01 | 3.81 | 1.44 | 2.21 | 2.08 | 2.22 | 1.30 | 1.18 | 0.98 | 1.18 | 1.42 | 0.29 | 0.39 | 0.28 |
| 5.00 | 3.18 | 3.62 | 3.71 | 3.63 | 1.23 | 2.12 | 2.01 | 2.13 | 1.81 | 1.37 | 1.29 | 1.36 | 1.80 | 0.50 | 0.63 | 0.47 |
| 5.25 | 2.69 | 3.36 | 3.36 | 3.39 | 0.99 | 1.99 | 1.88 | 2.01 | 2.29 | 1.62 | 1.63 | 1.60 | 2.15 | 0.76 | 0.93 | 0.72 |
| 5.50 | 2.26 | 3.05 | 2.99 | 3.08 | 0.77 | 1.83 | 1.70 | 1.86 | 2.72 | 1.93 | 2.00 | 1.90 | 2.45 | 1.06 | 1.26 | 1.01 |
| 5.75 | 1.90 | 2.71 | 2.61 | 2.74 | 0.58 | 1.65 | 1.50 | 1.68 | 3.09 | 2.28 | 2.38 | 2.25 | 2.69 | 1.37 | 1.58 | 1.33 |
| 6.00 | 1.60 | 2.35 | 2.25 | 2.38 | 0.43 | 1.45 | 1.29 | 1.48 | 3.38 | 2.64 | 2.75 | 2.61 | 2.87 | 1.69 | 1.89 | 1.64 |
| 6.25 | 1.36 | 2.00 | 1.91 | 2.04 | 0.31 | 1.23 | 1.08 | 1.27 | 3.63 | 2.99 | 3.09 | 2.95 | 3.00 | 1.98 | 2.17 | 1.93 |
| 6.50 | 1.15 | 1.68 | 1.60 | 1.71 | 0.22 | 1.03 | 0.87 | 1.06 | 3.84 | 3.31 | 3.40 | 3.28 | 3.10 | 2.24 | 2.41 | 2.20 |
| 6.75 | 0.97 | 1.39 | 1.32 | 1.42 | 0.16 | 0.84 | 0.70 | 0.87 | 4.01 | 3.60 | 3.67 | 3.57 | 3.16 | 2.45 | 2.61 | 2.42 |
| 7.00 | 0.82 | 1.13 | 1.08 | 1.16 | 0.11 | 0.67 | 0.55 | 0.70 | 4.16 | 3.85 | 3.91 | 3.83 | 3.21 | 2.63 | 2.76 | 2.60 |
| 7.25 | 0.68 | 0.91 | 0.87 | 0.93 | 0.08 | 0.54 | 0.44 | 0.57 | 4.29 | 4.07 | 4.12 | 4.05 | 3.24 | 2.77 | 2.89 | 2.75 |
| 7.50 | 0.57 | 0.73 | 0.70 | 0.74 | 0.06 | 0.44 | 0.35 | 0.46 | 4.41 | 4.26 | 4.29 | 4.24 | 3.25 | 2.88 | 2.97 | 2.85 |
| 7.75 | 0.46 | 0.57 | 0.55 | 0.58 | 0.05 | 0.37 | 0.29 | 0.39 | 4.50 | 4.41 | 4.44 | 4.40 | 3.26 | 2.94 | 3.03 | 2.93 |
| 8.00 | 0.37 | 0.44 | 0.43 | 0.45 | 0.04 | 0.33 | 0.26 | 0.35 | 4.58 | 4.54 | 4.55 | 4.53 | 3.26 | 2.98 | 3.06 | 2.97 |
| 8.25 | 0.29 | 0.34 | 0.33 | 0.34 | 0.04 | 0.32 | 0.24 | 0.34 | 4.64 | 4.63 | 4.65 | 4.62 | 3.25 | 2.99 | 3.07 | 2.97 |
| 8.50 | 0.22 | 0.25 | 0.24 | 0.26 | 0.04 | 0.33 | 0.25 | 0.35 | 4.69 | 4.70 | 4.72 | 4.69 | 3.24 | 2.97 | 3.05 | 2.95 |
| 8.75 | 0.17 | 0.19 | 0.18 | 0.19 | 0.04 | 0.37 | 0.28 | 0.39 | 4.72 | 4.74 | 4.76 | 4.74 | 3.22 | 2.92 | 3.01 | 2.90 |
| 9.00 | 0.12 | 0.14 | 0.13 | 0.14 | 0.05 | 0.43 | 0.33 | 0.45 | 4.74 | 4.75 | 4.78 | 4.75 | 3.20 | 2.83 | 2.94 | 2.81 |
| 9.25 | 0.09 | 0.10 | 0.10 | 0.11 | 0.06 | 0.51 | 0.40 | 0.53 | 4.75 | 4.74 | 4.77 | 4.74 | 3.17 | 2.72 | 2.85 | 2.70 |
| 9.50 | 0.07 | 0.08 | 0.08 | 0.08 | 0.08 | 0.60 | 0.47 | 0.62 | 4.75 | 4.70 | 4.74 | 4.70 | 3.13 | 2.59 | 2.74 | 2.57 |
| 9.75 | 0.05 | 0.07 | 0.06 | 0.07 | 0.09 | 0.69 | 0.55 | 0.71 | 4.73 | 4.65 | 4.70 | 4.65 | 3.09 | 2.46 | 2.63 | 2.43 |
| 10.0 | 0.04 |  | 0.05 |  | 0.11 |  | 0.61 |  | 4.71 |  | 4.65 |  | 3.05 |  | 2.53 |  |

Table 6. Bond Product Sum vs. pH for the non-carbonated system.

| BPS values for non-carbonated brine (Figure 7, in article file) | | | | |
| --- | --- | --- | --- | --- |
| pH Value | 1M CaCl_2_ | 0.01M CaCl_2_ | 1M NaCl | 0.01M NaCl |
| 2.00 | 0.22 | 5.08 | 0.54 | 6.69 |
| 2.25 | 0.38 | 6.83 | 0.91 | 8.74 |
| 2.50 | 0.65 | 8.45 | 1.48 | 10.41 |
| 2.75 | 1.09 | 9.88 | 2.29 | 11.69 |
| 3.00 | 1.76 | 11.10 | 3.37 | 12.66 |
| 3.25 | 2.74 | 12.11 | 4.69 | 13.38 |
| 3.50 | 4.06 | 12.93 | 6.18 | 13.92 |
| 3.75 | 5.73 | 13.57 | 7.74 | 14.32 |
| 4.00 | 7.70 | 14.08 | 9.29 | 14.61 |
| 4.25 | 9.85 | 14.47 | 10.76 | 14.83 |
| 4.50 | 11.96 | 14.79 | 12.09 | 15.00 |
| 4.75 | 13.73 | 15.09 | 13.26 | 15.13 |
| 5.00 | 14.92 | 15.45 | 14.27 | 15.25 |
| 5.25 | 15.44 | 15.93 | 15.14 | 15.35 |
| 5.50 | 15.37 | 16.46 | 15.86 | 15.47 |
| 5.75 | 14.89 | 16.83 | 16.44 | 15.64 |
| 6.00 | 14.23 | 16.87 | 16.89 | 15.92 |
| 6.25 | 13.55 | 16.53 | 17.15 | 16.31 |
| 6.50 | 12.99 | 15.91 | 17.20 | 16.68 |
| 6.75 | 12.57 | 15.17 | 16.99 | 16.82 |
| 7.00 | 12.29 | 14.45 | 16.52 | 16.60 |
| 7.25 | 12.11 | 13.85 | 15.89 | 16.07 |
| 7.50 | 12.01 | 13.39 | 15.20 | 15.37 |
| 7.75 | 11.95 | 13.05 | 14.55 | 14.64 |
| 8.00 | 11.92 | 12.79 | 14.02 | 14.00 |
| 8.25 | 11.90 | 12.56 | 13.60 | 13.52 |
| 8.50 | 11.88 | 12.32 | 13.29 | 13.18 |
| 8.75 | 11.86 | 12.07 | 13.05 | 12.96 |
| 9.00 | 11.81 | 11.82 | 12.87 | 12.83 |
| 9.25 | 11.74 | 11.56 | 12.72 | 12.75 |
| 9.50 | 11.61 | 11.31 | 12.60 | 12.69 |
| 9.75 | 11.39 | 11.08 | 12.50 | 12.66 |
| 10.00 | 11.05 | 10.86 | 12.42 | 12.62 |

Table 7. Bond Product Sum for the carbonated system.

| BPS values for carbonated brine (Figure 8, in article file) | | | | |
| --- | --- | --- | --- | --- |
| pH  Value | 1M  CaCl_2_ | 0.01M  CaCl_2_ | 1M  NaCl | 0.01M  NaCl |
| 2.00 | 0.21 | 1.49 | 0.50 | 1.53 |
| 2.25 | 0.37 | 2.33 | 0.83 | 2.38 |
| 2.50 | 0.63 | 3.42 | 1.36 | 3.49 |
| 2.75 | 1.05 | 4.73 | 2.13 | 4.81 |
| 3.00 | 1.72 | 6.16 | 3.17 | 6.24 |
| 3.25 | 2.69 | 7.61 | 4.47 | 7.68 |
| 3.50 | 4.03 | 8.96 | 5.94 | 9.02 |
| 3.75 | 5.75 | 10.17 | 7.50 | 10.21 |
| 4.00 | 7.80 | 11.21 | 9.02 | 11.24 |
| 4.25 | 10.01 | 12.12 | 10.45 | 12.13 |
| 4.50 | 12.19 | 12.96 | 11.78 | 12.95 |
| 4.75 | 14.08 | 13.54 | 13.03 | 13.53 |
| 5.00 | 15.47 | 14.20 | 14.18 | 14.16 |
| 5.25 | 16.29 | 14.93 | 15.19 | 14.88 |
| 5.50 | 16.56 | 15.63 | 15.96 | 15.58 |
| 5.75 | 16.36 | 16.14 | 16.40 | 16.11 |
| 6.00 | 15.83 | 16.30 | 16.42 | 16.28 |
| 6.25 | 15.13 | 16.05 | 16.04 | 16.05 |
| 6.50 | 14.43 | 15.46 | 15.38 | 15.47 |
| 6.75 | 13.84 | 14.71 | 14.61 | 14.72 |
| 7.00 | 13.40 | 13.94 | 13.88 | 13.95 |
| 7.25 | 13.08 | 13.27 | 13.27 | 13.27 |
| 7.50 | 12.86 | 12.73 | 12.80 | 12.72 |
| 7.75 | 12.69 | 12.30 | 12.44 | 12.28 |
| 8.00 | 12.53 | 11.94 | 12.13 | 11.91 |
| 8.25 | 12.36 | 11.58 | 11.83 | 11.55 |
| 8.50 | 12.17 | 11.19 | 11.50 | 11.14 |
| 8.75 | 11.96 | 10.71 | 11.11 | 10.66 |
| 9.00 | 11.73 | 10.13 | 10.62 | 10.07 |
| 9.25 | 11.46 | 9.44 | 10.04 | 9.36 |
| 9.50 | 11.18 | 8.73 | 9.42 | 8.64 |
| 9.75 | 10.90 | 8.05 | 8.82 | 7.96 |
| 10.00 | 10.65 |  | 8.29 |  |

Table 8 Bond Product Sum for the carbonated system using Song et al.’s ^27^ model with CO_2_ and mineral dissolution using alternative calcite surface stoichiometries.

| Song's SCM BPS values for Figure 9 in supplementary informaiton | | | | |
| --- | --- | --- | --- | --- |
| pH  Value | 1M  CaCl_2_ | 0.01M  CaCl_2_ | 1M  NaCl | 0.01M  NaCl |
| 2.00 | 1.82 | 2.05 | 1.78 | 1.97 |
| 2.25 | 1.87 | 2.26 | 1.82 | 2.20 |
| 2.50 | 1.95 | 2.55 | 1.88 | 2.50 |
| 2.75 | 2.09 | 2.92 | 1.95 | 2.86 |
| 3.00 | 2.31 | 3.36 | 2.06 | 3.29 |
| 3.25 | 2.65 | 3.84 | 2.20 | 3.75 |
| 3.50 | 3.13 | 4.33 | 2.37 | 4.22 |
| 3.75 | 3.79 | 4.81 | 2.57 | 4.68 |
| 4.00 | 4.61 | 5.26 | 2.78 | 5.11 |
| 4.25 | 5.51 | 5.69 | 2.99 | 5.51 |
| 4.50 | 6.42 | 6.09 | 3.21 | 5.89 |
| 4.75 | 7.21 | 6.35 | 3.42 | 6.15 |
| 5.00 | 7.81 | 6.65 | 3.62 | 6.43 |
| 5.25 | 8.19 | 7.00 | 3.79 | 6.76 |
| 5.50 | 8.33 | 7.37 | 3.92 | 7.11 |
| 5.75 | 8.28 | 7.68 | 3.99 | 7.41 |
| 6.00 | 8.08 | 7.85 | 3.97 | 7.58 |
| 6.25 | 7.80 | 7.86 | 3.89 | 7.60 |
| 6.50 | 7.51 | 7.74 | 3.75 | 7.49 |
| 6.75 | 7.27 | 7.55 | 3.59 | 7.30 |
| 7.00 | 7.09 | 7.34 | 3.45 | 7.10 |
| 7.25 | 6.98 | 7.17 | 3.34 | 6.93 |
| 7.50 | 6.92 | 7.05 | 3.26 | 6.82 |
| 7.75 | 6.90 | 7.00 | 3.21 | 6.76 |
| 8.00 | 6.91 | 7.00 | 3.19 | 6.75 |
| 8.25 | 6.96 | 7.04 | 3.18 | 6.80 |
| 8.50 | 7.04 | 7.13 | 3.17 | 6.88 |
| 8.75 | 7.16 | 7.25 | 3.18 | 6.99 |
| 9.00 | 7.29 | 7.38 | 3.18 | 7.10 |
| 9.25 | 7.44 | 7.52 | 3.17 | 7.22 |
| 9.50 | 7.58 | 7.59 | 3.16 | 7.27 |
| 9.75 | 7.70 | 7.61 | 3.13 | 7.27 |
| 10.00 | 7.79 | 5.70 | 3.10 | 4.92 |

Table 9 Brine chemistry of carbonated and non-carbonated brines in various ion type and salinity (This was computed using PHREEQC with consideration of calcite dissolution and water uptake of CO_2_ for carbonated brine)

|  | 1 mol/L CaCl_2_ | | 1 mol/L NaCl | | 0.01 mol/L CaCl_2_ | | 0.01 mol/L NaCl | |
| --- | --- | --- | --- | --- | --- | --- | --- | --- |
|  | non-carbonated | carbonated | non-carbonated | carbonated | non-carbonated | carbonated | non-carbonated | carbonated |
| H^+^ | 6.32×10^-9^ | 1.00×10^-4^ | 1.28×10^-10^ | 3.7×10^-5^ | 1.1×10^-9^ | 3.78×10^-5^ | 1.39×10^-10^ | 3.62×10^-5^ |
| Ca^2+^ | 9.69×10^-1^ | 9.43×10^-1^ | 4.34×10^-4^ | 9.43×10^-2^ | 1.00×10^-2^ | 6.28×10^-2^ | 1.45×10^-4^ | 5.68×10^-2^ |
| Na^+^ | - | - | 9.93×10^-1^ | 9.15×10^-1^ | - | - | 9.99×10^-3^ | 9.02×10^-3^ |
| Cl^-^ | 2.01 | 2.02 | 9.93×10^-1^ | 9.94×10^-1^ | 2.00×10^-2^ | 2.00×10^-2^ | 9.99×10^-3^ | 9.98×10^-3^ |
| CaHCO^3+^ | 6.68×10^-6^ | 1.06×10^-1^ | 1.25×10^-7^ | 3.67×10^-2^ | 8.95×10^-7^ | 3.35×10^-2^ | 1.11×10^-7^ | 3.19×10^-2^ |
| CaCl^+^ | 7.60×10^-2^ | 7.42×10^-2^ | 1.90×10^-5^ | 3.94×10^-3^ | 2.30×10^-5^ | 8.28×10^-5^ | 2.05×10^-7^ | 3.80×10^-5^ |
| HCO^3-^ | 2.78×10^-6^ | 4.54×10^-2^ | 1.07×10^-4^ | 1.51×10^-1^ | 1.38×10^-5^ | 1.39×10^-1^ | 9.68×10^-5^ | 1.45×10^-1^ |
| NaCO_3_^-^ | - | - | 1.05×10^-4^ | 4.62×10^-7^ | - | - | 1.07×10^-6^ | 5.03×10^-9^ |
| CO_3_^2-^ | 1.09×10^-7^ | 1.12×10^-7^ | 1.84×10^-4^ | 9.3×10^-7^ | 1.04×10^-6^ | 5.29×10^-7^ | 4.63×10^-5^ | 5.66×10^-7^ |

Table 10 Surface potential of oil-brine and brine-calcite in carbonated and non-carbonated brine (This is also computed using PHREEQC at the corresponding brines in Table 9)

|  | 1 mol/L CaCl_2_ brine | | 1 mol/L NaCl brine | | 0.01 mol/L CaCl_2_ brine | | 0.01 mol/L NaCl brine | |
| --- | --- | --- | --- | --- | --- | --- | --- | --- |
|  | non-carbonated | carbonated | non-carbonated | carbonated | non-carbonated | carbonated | non-carbonated | carbonated |
| pH | 8.21 | 4.02 | 9.85 | 4.91 | 9.05 | 4.87 | 9.92 | 4.91 |
| Surface Potential (oil-brine) (mV) | 14.55 | 56.72 | -80.68 | 19.32 | -31.33 | 20.90 | -83.00 | 19.03 |
| Surface Potential (brine-calcite) (mV) | 63.76 | 64.10 | -7.12 | 37.17 | 41.15 | 40.09 | -9.46 | 38.27 |
